# Self-medication with anti-malarials is a common practice in rural communities of Kilosa district in Tanzania despite the reported decline of malaria

**DOI:** 10.1186/1475-2875-13-252

**Published:** 2014-07-03

**Authors:** Beatrice Chipwaza, Joseph P Mugasa, Iddy Mayumana, Mbaraka Amuri, Christina Makungu, Paul S Gwakisa

**Affiliations:** 1 Nelson Mandela African Institute of Science and Technology, P.O. Box 447, Arusha, Tanzania; 2 Ifakara Health Institute, P.O. Box 53, Ifakara, Tanzania; 3 National Institute for Medical Research, Amani Medical Research Centre, P.O. Box 81 Muheza, Tanga, Tanzania; 4 Jhpiego, P.O. Box 9170, Dar-es-Salaam, Tanzania; 5 Genome Science Centre and Department of Veterinary Microbiology and Parasitology, Sokoine University of Agriculture, P.O. BOX 3019, Morogoro, Tanzania

**Keywords:** Self-medication, Rural communities, Anti-malarials, Kilosa, Tanzania

## Abstract

**Background:**

Self-medication has been widely practiced worldwide particularly in developing countries including Tanzania. In sub-Saharan Africa high incidences of malaria have contributed to self-medication with anti-malarial drugs. In recent years, there has been a gain in malaria control, which has led to decreased malaria transmission, morbidity and mortality. Therefore, understanding the patterns of self-medication during this period when most instances of fever are presumed to be due to non-malaria febrile illnesses is important. In this study, self-medication practice was assessed among community members and information on the habit of self-medication was gathered from health workers.

**Methods:**

Twelve focus group discussions (FGD) with members of communities and 14 in-depth interviews (IDI) with health workers were conducted in Kilosa district, Tanzania. The transcripts were coded into different categories by MaxQDA software and then analysed through thematic content analysis.

**Results:**

The study revealed that self-medication was a common practice among FGD participants. Anti-malarial drugs including sulphadoxine-pyrimethamine and quinine were frequently used by the participants for treatment of fever. Study participants reported that they visited health facilities following failure of self-medication or if there was no significant improvement after self-medication. The common reported reasons for self-medication were shortages of drugs at health facilities, long waiting time at health facilities, long distance to health facilities, inability to pay for health care charges and the freedom to choose the preferred drugs.

**Conclusion:**

This study demonstrated that self-medication practice is common among rural communities in the study area. The need for community awareness is emphasized for correct and comprehensive information about drawbacks associated with self-medication practices. Deliberate efforts by the government and other stakeholders to improve health care services, particularly at primary health care facilities will help to reduce self-medication practices.

## Background

Self-medication can be defined as the use of drugs to treat self-recognized disorders or symptoms or the intermittent or continued use of a prescribed drug for chronic or recurrent diseases or symptoms without the advice of a physician [1]. Self-medication is commonly practiced world-wide particularly in developing countries [2-5]. It is considered as an alternative way for people who cannot afford the cost of health care services. Also due to lack of access to health care facilities many individuals opt to receive initial treatment for febrile illnesses at home using herbal medicines, oral antipyretics, anti-malarial or antibiotic drugs purchased in local shops without prescription [6]. Self-medication should be distinguished from home-treatment in the sense that, self-treatment is used when individuals treat themselves without the direct involvement of another person concerning their illness. Home-treatment is the provision of treatment by formal or informal caregivers in the home, for instance home-based management of malaria [7] as opposed to treatment given at a recognized health care facility.

Risks associated with self-medication include lack of clinical evaluation of the condition by a health care provider which could result in misdiagnosis and incorrect choice of drugs, delays in seeking appropriate treatments, use of excessive drugs or lower dosage and prolonged duration of use [8]. Other potential risks include the development of adverse drug reactions, dangerous drug interactions and masking of a severe disease [9]. The other concern with self-medication practice is the risk of developing resistant pathogens. The current problem of antimicrobial resistance and the development of parasite resistance to anti-malarial drugs that has been reported in several countries are likely to be associated with irrational use of antibiotics and anti-malarial drugs [10,11].

For many years, malaria has been the leading cause of fever in sub-Saharan Africa [12,13]. In the year 2000, malaria accounted for over 43% of outpatient attendance, 42% of all hospital admissions and 32% of hospital deaths in health facilities in Tanzania [14]. Self-medication with anti-malarial drugs has been widely practiced in different countries including Tanzania [4,15]. In recent years, there has been a reduction of malaria prevalence particularly in malaria endemic countries [16-19]. Although there are reports on decline in malaria transmission, morbidity and mortality, fever is still a major complaint in many outpatient clinical settings [20], which points out to importance of non-malaria febrile illnesses [21,22]. Non-malaria febrile illnesses such as typhoid fever, urinary tract infections (UTI), acute respiratory tract infections (ARIs), rotavirus infection, brucellosis, tick-borne relapsing fever, leptospirosis, dengue fever and Chikungunya virus infection have been reported in Tanzania [22,23]. With the decrease of malaria cases there is an escalating trend towards self-medication used as a best guess for treating febrile cases in the absence of laboratory diagnosis or any undiagnosed febrile illness [24]. Accordingly, understanding the patterns of self-medication, especially with anti-malarials, during this period when most instances of fever are presumed to be due to non-malaria febrile illnesses is important. However, few studies on self-medication practice have been conducted in Tanzania [4,15]. In this study, self-medication practice among community members was assessed and information was gathered from health workers from primary health facilities and district hospital on the habit of self-medication among community members.

## Methods

### Study area

The study was conducted in Kilosa district, which is one of the six districts of the Morogoro region of Tanzania. The district has an area of about 14,245 square kilometres. The district is bordering Dodoma and Iringa regions in the west, Mvomero district in the east, Kilombero district in the south and Tanga and Arusha regions in the north. The district lies between 6°S and 8°S, and 36°30′E and 38°E. Based on 2012 population census, Kilosa district has 438,175 people of whom 218,378 are males and 219,797 are females. The proportion of children under the age of 5 and 10 years is 65,654 and 62,235 respectively [25]. The predominant ethnic groups are Kaguru, Sagara and Vidunda. The major economic activities in Kilosa district are crop production and livestock keeping. Kilosa district is comprised of nine divisions and 37 wards, 164 villages and 1,030 hamlets [26] with Kilosa town being its administrative headquarter. For health care services, the district has 71 health facilities; among those three are hospitals, seven health centres and 61 dispensaries [27]. In Tanzania, dispensary is the primary level health care facility which provides basic curative care. A dispensary caters for 5,000 people and oversees all the village health services. Health centre is the second level facility, which offers outpatient and inpatient services and caters for 50,000 people and supervises all the dispensaries in the Division [28]. Hospitals are third level facilities, however, in term of hospital levels, district hospital is on level one and act as referral for the primary health care facilities in the district [28].

Kilosa district is an area with holoendemic malaria transmission with seasonal peaks following the long and short rainy seasons [29]. In 2007, malaria accounted for about 55.5% of the total outpatient cases, and 60% of the total deaths among under-fives admitted to the health facilities [30]. However, based on Tanzania HIV and Malaria Indicator Survey, malaria prevalence in Morogoro region was estimated to be 15.7% in 2007–2008 [31] and decreased to 13% in the year 2011–2012 [32]. The district does not have any active home management of malaria program or community case management. There were 152 pharmacies/drug shops in the district and most of them were located in urban and semi-urban areas [33].

### Study design and sampling procedures

**Figure 1:**
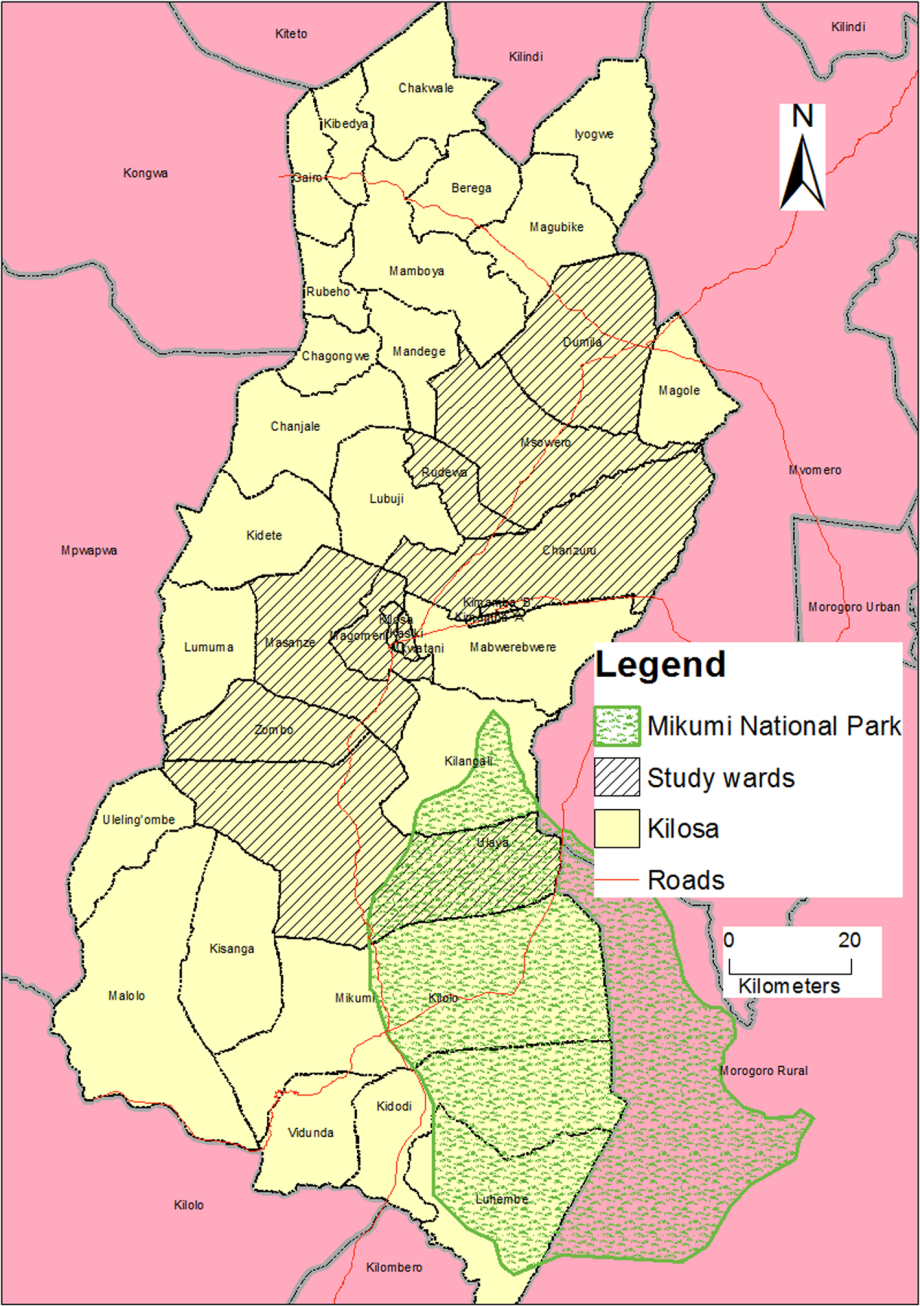
Map of Kilosa district showing the study wards.

In the present study, FGDs and IDIs were conducted with community members and health workers respectively. The participants for the FGD were caregivers of children aged less than 10 years. This group of participants was targeted because children at this age are considered to be susceptible to illnesses [22,34]. The FGDs were specifically conducted in Kasiki, Magomeni, Chanzuru, Masanze, Rudewa and Kimamba wards and the participants were recruited from urban, peri-urban and rural areas with the assistance of local government and village leaders. For IDI, health workers who were responsible for medical consultations at the facilities were eligible to participate into IDI. Health workers were recruited from health facilities located in Dumila, Chanzuru, Ulaya, Zombo, Kilosa, Magomeni, Msowero, Kimamba, and Mkwatani wards. These wards were purposively selected based on (i) geographical representation within the district (Figure 1), (ii) the presence of government health facilities, (iii) connectivity of the wards and ease of accessibility by road.

### Data collection

A topic guide for FGD and IDI consisted of semi-structured questions corresponding to the research questions was developed. The guide consisted of questions about the magnitude of self-medication practice, categories of drugs commonly self-prescribed, indications for self-medications and reasons for self-medications. The topic guide was prepared in English and then translated to Swahili, which is the most widely spoken language of the community. All discussions and interviews were done with the assistance of a note taker and a moderator who is experienced in conducting FGDs and IDIs. In addition to taking notes, conversations were recorded by digital recorder. A total of 12 FGDs, five FGDs with men and seven FGDs with women were conducted. Each FGD consisted of about 6–8 people and during the discussions men and women were interviewed separately to allow participants to talk freely. Two FGDs were held per day and each FGD took about 90 to 120 minutes. In-depth interviews with health workers on average took one hour and a maximum of two health workers were interviewed from each health facility. Only one health worker was interviewed at a time and no health worker refused to participate in the study.

### Data management and analysis

All FGDs and IDIs were transcribed verbatim and translated from Swahili to English. The transcripts were converted into rich text format and imported into MaxQDA (version 11, VERBI Software). Text files were independently reviewed by the two researchers (IM and CM) before agreeing on the different themes and categories. When there was differing interpretations, the discussion between the researchers took place until the consensus was reached. The findings were also validated by the interviewing researcher (BC). The agreed themes and categories were then coded and the retrieved segments were analysed using thematic content analysis and their respective codes were exported to Excel for quantitative analysis.

### Ethics statement

Ethical approval was obtained from Institutional Review Board of Ifakara Health Institute (IHI/IRB/No: 01–2013) and Medical Research Coordinating Committee of Tanzania’s National Institute for Medical Research (NIMR/HQ/R.8a/Vol.1X/1472). Health workers and the FGD participants were briefed on the aims and objectives of the study and a written informed consent was obtained from those who were willing to participate in the study. To protect identification of the respondents and FGD participants, all personal information that could identify the study participants were only used during the analysis and omitted from the final reports. The participants were assured of anonymity in the presentation and publishing of the data.

## Results

A total of 93 participants were interviewed during FGDs, among whom 56 were women and 37 were men. Many FGD participants were subsistence farmers and the majority had primary level of education (Table 1). Twelve health facilities were visited, among them were one district hospital, two health centres and nine dispensaries. Fourteen health workers were interviewed, including eight clinical officers, one assistant medical officer, three nurses and two medical attendants. Among the 14 health workers, six were males and eight were females; eleven health workers (11/14) had work experience ranging from 1–20 years whereas 3 (3/14) had experience ranged from 21–40 years. This study identified four major themes and subthemes as indicated in Table 2.

**Table 1:** Demographic characteristics of FGD participants

**Category**	**Subcategory**	**n**	**%**
Sex	Male	37	39.8
	Female	56	60.2
Age	18-30 y	41	44.1
	31-60 y	52	55.9
Occupation	Subsistence farming	87	93.5
	Business	1	1.1
	Employed	5	5.4
Education level	Illiterate	11	11.8
	Primary	73	78.5
	Secondary	7	7.5
	Tertiary	2	2.2

**Table 2:** Summary of the key findings

**Key themes identified**	**Subthemes**
**Self-medication practices**	The magnitude
	Perception
	Targeted group
**Categories of drugs and indications**	Anti-malarial drugs for treating fevers
	Antipyretics/pain killers for fever and pain
	Antimicrobials for diarrhoea, coughing and wounds
	Antihelmintics for loss of appetite
**Outcomes of self medication**	Seek treatment from health facility following treatment failure
	Change of the drug following treatment failure
**Reasons for engaging in self-medication**	Shortages of drugs at health facilities
	Long waiting time at health facilities
	Long distance to health facility
	Inability to pay for health care costs
	Freedom to choose the drug of their choice

### Self-medication practices

Self-medication was found to be a common practice among FGD participants. When FGD participants were asked about the habit of self-medication without medical advice, the vast majority reported that the practice of self-medication was very common in the community. Self-medication was mostly practiced for children even though other participants admitted buying drugs for themselves. During the discussions, only a minority of the participants said that they were not self-medicating. It was noted that the use of non-prescription drugs was equally practiced by women and men across all groups regardless of their age. In addition, findings revealed that self-medication was practiced by FGD participants from wards quite near health facilities as well as those that are far off from health facilities. Across the focus groups, participants explained that once their children get fever, they preferred to purchase drugs from the pharmacies/drug shops or use left-over medicines from their homes, neighbours, relatives or friends. It was reported that in most cases, the parents/guardians decided on what drug to use even though few participants commented that they sought advice from vendors. They further said this habit was very common among members of the community and they do so very often. During all FGD sessions, the participants pledged their allegiance to this behaviour and they considered it as the norm for health seeking practice. On the other hand, although they agreed being engaged in such practices, few participants perceived self-medication as unsuitable way of seeking health care and recognized that they should seek treatment from health facilities. However, the majority of participants regarded self-medication as an easier way to obtain prompt treatment. They further said they considered it as an alternative solution to the predicament of public health care services.

“This habit is very common, when a person feels sick or their children are sick, we normally run to a pharmacy without consulting a medical doctor” (FGD12-men, P6).

“More than half of the village members depend on the pharmacies” (FGD 8-women, P1).

“In short that habit exists in our community. I also buy drugs for my children without medical advice” (FGD 11-men, P3).

Interviewed health workers were asked to give their views regarding the habit of self-prescribing behaviour among community members, and almost all of them acknowledged the existence of self-medication behaviour. Health workers explained that it was only a few individuals who bring their children to health facilities but most of them opt to buy drugs from the pharmacies or drug shops without prior medical advice. Health workers insisted that the community is used to practice self -medication for many years and if no preventive measures are taken, the habit will keep growing and very likely will lead to the development of resistant pathogens or microbes.

“Absolutely yes, it happens a lot! Most of them would go to the pharmacy instead of coming here. Normally, a few individuals would come for prescriptions but the majority of them don’t” (IDI-clinical officer, R11).

“A few individuals bring them here once a child gets ill, most parents go and buy medicines from a pharmacy” (IDI-registered nurse, R1).

### Categories of drugs and indications for self-medication

When it came to indications for self-medication and type of drugs they use, among the various indications (symptoms/signs) reported by the participants, fever was the most common, followed by diarrhoea, coughing, loss of appetite and flu. Few participants listed symptoms such as abdominal pain, wounds and headache.

“When my children get fever, I normally go to a drug shop and buy medicine for them” (FGD 5-women, P8).

“There are some people who simply go to the pharmacy and buy medicines as their children become hot” (FGD 7-men, P1).

With regards to category of drug products that were commonly self-administered, anti-malarials were frequently mentioned by majority of the participants followed by antipyretics (pain killers), antimicrobials and antihelmintics. However, this study revealed that participants’ preferences on the choice of anti-malarial drugs were varying. Majority of participants reported using artemether-lumefantrine (ALu), but drugs which are no longer used as first-line treatment, such as sulphadoxine-pyrimethamine (SP), and drugs reserved for second-line therapy (quinine) were also used. During the day of discussion, two participants admitted that their children were under anti-malarial treatment and that the drugs were purchased from drug shops without medical advice.

“I’m one of them. I prefer using ALu. Even today, I bought ALU and now my child is under anti-malarial treatment” (FGD 11-women, P4).

“It’s true what she says. We buy quinine from the pharmacy for our children” (FGD8-women, P6).

It was also revealed that self-medication with anti-malarial drugs was common following self-diagnosis of fever. The participants explained using anti-malarial drugs for the treatment of fever since many believed that fever is caused by malaria.

“.... They normally buy ALu and panadol. ....when they see their children have fever, they think it’s malaria; they just go to the pharmacy and buy medicines for their children” (FGD 12-men, P1).

Similarly, interviewed health workers reported that several members of the community self-medicated most frequently with anti-malarial drugs although antimicrobials were occasionally used. Health workers explained that many community members use anti-malarial drugs particularly when their children get fever. Patients would demand drugs such as SP or quinine, which are not prescribed as first line treatment against uncomplicated malaria. They further pointed out that some patients do not believe that ALu can cure malaria. Health workers said even though they do not prescribe SP, patients obtain such drugs from the pharmacies/drug shops, which signify irrational use of anti-malarial drugs. This is a chronic problem in the community and they suggested that necessary measures should be taken to stop this habit. In addition, health workers insisted on the need for creation of awareness of other causes of fever among community members.

“The habit of self-medication is common in our community. Yesterday, I was told that the child was given amodiaquine syrup but the problem persisted. Another one was given ALu and finished the course of treatment without improvement. This morning, one patient told me that his child was given quinine, but the child was so weak and they decided to bring him to the dispensary, then I found that the child had pneumonia” (IDI-clinical officer, R8).

“On its part, the community should be made aware of the medical conditions which cause fever as many believe that fever is synonymous to malaria; and some run to a pharmacy and get SP and for their children. But if we educate members of the public, we’ll make them think of health care services” (IDI-Clinical officer, R8).

“Sometimes you prescribe ALu, you may find they tell you that, ‘I don’t need ALu, I need metakelfin (SP) and that’s when they go and buy metakelfin” (IDI-clinical officer, R6).

“They say that, they believe malaria is cured by quinine injection; so once they have bought it, they bring to you for you to inject them....” (IDI-clinical officer, R13).

The other category of drugs that was found to be self-prescribed by a considerable number of the participants was antipyretics/pain killers, such as paracetamol and aspirin. However, it was noted that in most cases, antipyretics were applied together with anti-malarial drugs. While participants believed that antipyretics alone could not cure fever, some reported self-prescription with antimicrobials, such as metronidazole (Flagyl®), co-trimoxazole (Septrin®) and amoxicillin. Flagyl was the most commonly reported antibiotic and it was used by the majority of participants when their children had diarrhoea. Antimicrobials were also used for treatment of cough and wounds. Furthermore, few participants admitted using antihelmintics when their children had loss of appetite.

“In most cases, we like buying panadol and ALu when we find our babies are hot” (FGD2-men, P5).

“You can try to offer them flagyl, which many people use to prevent children from diarrhoea” (FGD 11-men, P6).

“If my child losses appetite, and then I go to the hospital, and I do not get mebendazole tablets, I therefore, normally go to the pharmacy” (FGD 10-women, P1).

### The outcome following self-medication

This study found that most FDG participants visited health facilities following failure of self-medication or if there was no significant improvement after self-medication. However, few participants reported to opt for change of drug, for instance, quinine instead of ALu which was initially applied. While majority of participants mentioned treatment failure commonly encountered after self-medication, only few participants confirmed that sometimes they got better following self-treatment. The participants admitted that it was not unusual to experience persistence of fevers or recurrence of fever among children who were self-medicated. They further explained that in case there was persistent fever or recurrence of fever, they preferred taking their children to a health care facility.

“If the hospital is distant, I simply buy medicines for the children. But if the condition gets worse, I take them to the health facility” (FGD 10-men, P5).

“Personally, once I see the child has high temperature, I know this child has got malaria. I can simply buy some tablets in line with his fever and give him. But if the fever recurs after taking tablets, I rush him to hospital” (FGD 11-women, P1).

Interviewed health workers acknowledged that sometimes they receive patients (children) who were in a critical condition. Sometimes caregivers admitted that delays to bring children to health facilities were often caused by initial use of anti-malarials at home, and decision to bring the child to a health facility when fever persisted. Health workers emphasized the need to prevent self-medication practice as it delays prompt and accurate treatment. They further insisted the necessity of obtaining medical consultation before using medicines, since not all fevers are due to malaria as it was perceived by community members. Health workers further pointed out that the delays in seeking treatment from health facilities and mismanagement of febrile patients would continue causing unnecessary deaths if the habit is not stopped. They also concluded that self-prescriptions in most cases do result into improper treatment of febrile illnesses.

“Children from the age of six months to five years are the ones who are mostly brought here after they have gone to pharmacies without getting improvement and we get some who are already very serious. Sometimes you prescribe the artemisinin-based combination therapies (ACT) for them; they would say ‘Oh, you have prescribed ALu for me? I have already taken much of it” (IDI-Clinical officer, R11).

“When they see their children have fever, they simply go to pharmacies to buy ALu for their children. If you see them coming here, you simply know that things haven’t gone well since most of them like going straight to pharmacies” (IDI-Nurse, R3).

### Reasons for engaging in self-medication

In all focus groups, the participants reported a range of factors that prompt them to self medication. However, the common listed reasons were shortages of drugs at health facilities, long waiting time at health facilities, long distance to the health facility, inability to pay for health care costs and the freedom to choose the drug of their choice. The majority of the participants raised concerns about drug shortages at most health care facilities. The participants discussed how they spend long time at health facilities waiting to get consultations from medical doctors and thereafter only to be told that the prescribed drugs were not available and hence they should buy from the pharmacies/drug shops. One of the FGD participants said:

“Let me just add that the issue of shortages of drugs in health facilities disturbs us most. We go to the health centre and queue up for the service till 2 pm and then you are told to go to the dispensing window to get the prescribed medicine, on reaching there, you find there are no drugs and hence you should go to buy the same from somewhere else. Therefore, it’s better to buy drugs from the pharmacies rather than going to the health centre” (FGD 9-men, P6).

The problem of drug shortages was also reported by the majority of interviewed health workers as an ongoing problem. Health workers admitted receiving inadequate supply of drugs from the government (Medical Store Department), which does not meet the demand of the community. Health workers further pointed out that the absence of drugs contributes to self-medication practices.

“Sure, they used to do so very often. They believe that there are no drugs at the dispensary, so they think if they come here, they would be told to go and buy the drugs. Instead, they decide to go straight to the pharmacy” (IDI-clinical officer, R8).

The majority of the participants and some interviewed health workers spoke about long waiting time at health facilities as a contributor to self-medication. The FGD participants and health workers reported that the issue of staff shortages, particularly clinicians contributes to delays to obtain medical advice. The participants complained about queuing for many hours before getting medical consultation. Further, they said that their sick children may progress to severe illness during the waiting period and they expressed fear of losing their children due to delays in receiving treatment. The participants assumed that the easier alternative way is going straight to the pharmacy.

“They decide to do so because of the scarcity of staff to attend them. When they come for medical care, they find themselves queuing for quite long as a result of few staff; they think the shortcut is to buy drugs from a pharmacy rather than waiting for service for many hours at health facility. I think this is the main reason” (IDI-clinical officer, R2).

The absence of nearby health facilities and inability to pay for health care costs emerged as among the reasons for the participants to engage themselves into self-medication practice. The issue of long distance was reported mostly by the participants who lived far from health facilities. During the interviews with health workers, some explained that majority of community members live in remote areas where there is lack of access to health care facilities. In addition, a large number of participants admitted that they could not afford health care costs.

“I am one of them, as you know there is no dispensary here; you must go to [name of dispensary].... I don’t have money to cover the travel cost, So I must spend the little amount I have to buy medicines from the pharmacy and give to the baby” (FGD5-women-P4).

“If you go to hospital, you are told to buy syringes which cost 500/-Tanzanian Shillings (US $ 0.3) each. So I can’t pay that amount every time I go there; I better go straight to [name of the drug shop] and get the drug for my child” (FGD 4-women, P7).

The participants preferred self-medication practice due to freedom of choosing the drug of their choice. Some participants said that if they go to the pharmacy/drug shop, they were able to make the decision on which drug to use. Also, they admitted going to the pharmacy/drug shop because they could obtain drugs such as SP, quinine and antihelmintics, which they believed would cure their sick children but these drugs were rarely prescribed from health care facilities.

“At the pharmacy the vendor would ask to know the type of drugs you normally use, and you mention quinine or homaquine. They give you the medicine and explain the dosage for your child” (FGD 10-men, P4).

“....when you go to a pharmacy, you can obtain drugs such as SP, Amodiaquine but if you go to the health facility, you can’t be given such drugs” (FGD 9-men, P8).

## Discussion

The findings from this study have revealed that self-medication was indeed a very common practice among the study participants and they use to treat self-recognized symptoms. Self-medication was equally practiced by men and women across all FGDs. Similar finding have been reported from other studies in Tanzania where the majority of participants self-medicated [4,35]. The use of non-prescribed drugs was common even in places where health professionals were easily accessible. Evidence from various studies including the current study has shown that self-medication was commonly practiced in children [36,37]. The present study showed that in most cases parents/guardians were the ones who made diagnosis for their children’s illnesses and decided on which drugs to use and only a few participants admitted to seek medical advice from vendors before purchasing drugs. With regards to sources of drugs, the majority obtained drugs from pharmacies/drug shops whereas other participants used left over medicines at home or from neighbours, relatives or friends which is in agreement with studies conducted in Kenya and Nigeria [38,39]. Interviewed health workers from the current study expressed their concerns on self-medication practice among community members and they perceived it as a chronic problem. They suggested that appropriate measures should be taken to stop this behaviour.

Anti-malarial drugs were the most frequently used medicines by the majority of the participants, followed by antipyretics, antimicrobials and antihelmintics. In Tanzania, high levels of chloroquine resistant *Plasmodium falciparum* malaria led to the change of treatment guidelines for malaria in 2001, where the first-line drug chloroquine was replaced by SP [40]. However, SP remained the first-line until it was replaced by ALu in 2006 and quinine was used as second-line as well as the drug of choice for severe malaria [41]. Meanwhile, SP remains to be used as a drug of choice for intermittent presumptive treatment for malaria in pregnancy (IPTp). Although majority of the participants from this study were self-medicating with ALu following the occurrence of fever, others were using ineffective anti-malarials (SP) or second-line drugs (quinine). These findings correlate with results from a previous study in Kenya where ACT was the drug of choice for a large percentage of participants who were self-medicated followed by SP, amodiaquine, choloroquine and quinine [38]. Similarly, evidence from the various studies indicate that self medication with anti-malarials appears to be a common practice in other countries such as Ghana, Ethiopia, Sudan, Cameroon and Nigeria [42-44]. Treating all fever patients with anti-malarial drugs can lead to drug wastage hence a potential for drug shortage, failure to treat the actual cause of fever (non-malarial febrile illnesses) and unnecessary drug side effects. In addition, inappropriate prescription and dispensing of anti-malarials may increase the risks of developing parasite resistance [11]. Currently, quinine is the effective drug and is used for treatment of severe malaria however, some of the participants from the present study were self-prescribed with quinine and they believed to be cured by this drug. If this wrong perception is not corrected, the misuse of anti-malarial drugs will pose a challenge to the achievements attained so far, whereby the reduction of malaria is due to successful control efforts including the use of effective anti-malarial drugs [45].

With regards to indications for self-medication, the findings from this study have shown that the most common illnesses that influenced the participants to self-medicate were fever, followed by diarrhoea, coughing and loss of appetite. Fever is the major complaint, which was also raised from other reports even though other symptoms (headache, cough and diarrhoea) were not in the same order as shown by the present study [2,46,47]. This study has demonstrated that the concept that fever is caused by malaria still exists among community members. In addition, this was acknowledged by the interviewed health workers, who also emphasized on creation of awareness on non-malaria febrile illnesses. The findings from this study revealed that in most cases antipyretics were applied with anti-malarials, which agrees with findings from Nigeria where fever was associated with anti-malarial drugs and antipyretics [46]. Furthermore, it was noted that antimicrobials (metronidazole) and antihelmintics were used for the treatment of diarrhoea and loss of appetite, respectively. Diarrhoea is a clinical manifestation of several illnesses which are caused by different aetiological agents [48]. Therefore, the use of metronidazole for treatment of diarrhoea was inappropriate. Similarly, the belief that loss of appetite is caused by only worms was unrealistic since several other illnesses could also show a similar symptom.

In the present study, it was found that majority of the participants sought treatment from health care facilities if self-medication failed. Study participants explained that when they encountered persistence or recurrence of fever, they usually opted for treatment in a health care facility. These findings concur with observations reported in studies from Ghana and Sudan, where a large percentage of participants who were self-medicated had treatment failure and among those, the majority consulted physicians at health care facilities [49,50]. The present study emphasizes on concerns of health workers, on the occurrence of avoidable deaths due to treatment delays and mismanagement of febrile patients particularly children. It was clearly observed that, community members self-medicated with anti-malarials for the purpose of treating fevers, at the same time, evidence has shown a reduction of incidence of malaria [21]. Moreover, in Tanzania, recent studies have indicated the importance of non-malaria febrile illnesses and there is clear evidence that most fevers are non-malarial [22]. Treatment failure observed in this study may be due to mistreatment of non-malaria febrile illnesses. It is worth commenting that besides the risks of developing parasite resistance due to misuse of anti-malarial drugs, in most cases febrile patients do not receive the right drug for the right illness.

Self-medication, as per opinions by study participants, appeared to be driven by several factors. Shortages of drugs in most health facilities have been a major problem for a long time as it has been pointed out from other studies in Tanzania [51,52]. Stock-out of drugs was marked by interviewed health workers from this study as a major factor for self-medication. Also, long waiting time was also mentioned as a contributor to self-medication and was mainly due to shortages of health workers, particularly clinicians. Long waiting time at health facilities was equally reported by Eldalo in 2013 being one of the reasons for self-medication [50]. The shortage of health workers in Tanzania remains to be a challenge [53] and contributes to long queues at health facilities. Furthermore, access to health facilities was also a major concern. The rapid growing population particularly in rural areas is not in proportional with the established primary health care facilities. The number of villages in Kilosa district exceeds the number of health facilities, therefore, many primary health facilities serve more than one village. It was also observed that study participants were not able to pay for the cost of health care and hence practiced self-medication, which they believed to be a cheaper option. This finding agrees with studies done in Kenya, Sudan and Mali [37,38,50] and suggests that provision of affordable medical services will assist in reducing self-medication practices. The study also revealed that some participants preferred self-medication because purchasing drugs from pharmacies/drug shops gave them an opportunity to obtain drugs of their choice. Surprisingly, there were participants who believed that only certain drugs could cure their children’s illnesses. During the interviews, health workers explained that some patients would demand for prescription of certain drugs, such as quinine or SP, and if not given they will obtain those drugs from pharmacies. This means that any strategies for prevention of self-medication should take into consideration the correction of these wrong beliefs among community members.

### Strengths and limitations

FGD participants were selected from different divisions, wards and hamlets and thus hence their views were a representation of the general population in the district. Interviewed health workers were selected from different health care facilities levels (dispensary to district hospital) to obtain wide scope of views of health care staffs. More FGDs were conducted with women as compared to men, because women are perceived to be responsible for childcare in the family. However, the results from this study showed no differences in self-medication practices between women and men. It is also possible that some information was lost during the translation of the transcripts before analysis.

## Conclusion

This study demonstrates that self-medication practice is very common among rural community members. This indicates that there is a need for the community members to get access to correct and comprehensive information about the drawbacks associated with self-medication practices. A national commitment to solving the problem of irrational use of medicines is urgently required. This would require strengthening of the regulatory control for dispensing of drugs particularly anti-malarials and antimicrobials. On the other hand, massive health education aimed at behavioural change and creation of awareness on non-malaria febrile illnesses is recommended.

## Abbreviations

UTIs: Urinary tract infections; ARIs: Acute respiratory tract infections; FGDs: Focus group discussions; IDIs: In depth interviews; ALu: Artemether-lumefantrine; SP: Sulphadoxine-pyrimethamine; ACT: Artemisinin-based combination therapy; IPTp: Intermittent presumptive treatment for malaria in pregnancy; COSTECH: Tanzania commission for science and technology.

## Competing interests

The authors declare that they have no competing interests.

## Authors’ contributions

BC and PSG conceived and designed the study. BC carried out the data collection in Kilosa district and validated the findings. IM and CM performed the analysis. BC drafted the manuscript whereas PSG, JPM, IM and MA contributed to manuscript preparation. All authors read and approved the final manuscript.
